# A Memorable Ceremony at Guy's Hospital

**Published:** 1920-02-07

**Authors:** 


### THE PRESENTATION TO SIR E. COOPER PERRY.

### A Memorable Ceremony at Guy's Hospital.

A ceremony, short but most impressive to all who hold Guy's and its wonderful traditions dear, was held in the Court room of the hospital on Thursday, January 29, at 4 p.m., when formal presentation was made to Sir Cooper Perry of tokens of appreciation for his long and magnificent service and devotion to his duties as Superintendent, from which on January 31 he would retire to pass on to the appointment of Principal Officer to the University of London. It is a matter of congratulation that whilst thus losing a great man's services as Superintendent, Guy's will retain the advantage of his advice and wide experience, though in a new capacity as a Governor of the Corporation, Sir Cooper having been elected to that body at the last meeting of the General Court.

For some weeks previous arrangements had been made in practically every section of Guy's, from the medical staff to the works department, that subscriptions, limited in individual amount, but gladly given by all, should be collected. Each subscriber, and there were indeed hundreds, supplied his or her signature to be inserted in a beautifully bound and illuminated book headed by the very appropriate address which we publish later. This volume, which must be a delightful possession to one who has been so much and so close a friend to the community it represents, is later to be presented informally to Sir Cooper, but at the ceremony itself the silver tokens purchased from the actual subscriptions were arranged on the Court room table. In so crowded a gathering they may have seemed but trifling things to the casual observer, but to the givers and, we know, to the "Super" himself they represented all that unspoken affection and an appreciation impossible to magnify that is felt by Guy's for its famous and unequalled Sir Cooper Perry.

Briefly we will enumerate these gifts and then pass on to our impression of this notable gathering in which we were privileged to find ourselves. First there was a silver tea-set from the staff of the hospital and schools, including consultants and the past and present students both medical and dental. From the administrative and office staff came a pair of silver candelabra and a cake basket. The nursing staff gave a very fine clock, and the works department staff a large silver cigar-box, which, as was humorously suggested, would prove an ever-present joy to the recipient—a non-smoker, we believe—in providing yet further perfection in his already renowned hospitality.

A few minutes before the time appointed, the usual afternoon quiet of the colonnade and quadrangle was disturbed by an ever-increasing stream of men and women towards the Court room. From the wards, from the theatres, from every part of Guy's came every doctor, nurse and student who could possibly leave their work for that great half-hour. Guy's Statue looked down on a truly exceptional collection of staff cars lined up below him and yet more continued to arrive. In the Court room itself the crowd grew denser every minute. There was no seating, no definite position, but each section automatically arranged itself until one saw one of the most representative collection of the Guy's community it has been our privilege to witness. Practically to a man the senior and junior staff were present, and many a face, for years now strange to the Southwark hospital, was seen in that surging throng. By four o'clock not another person could be squeezed in, the landings and stand below were equally packed, and all waited in quiet expectation for the "Super's" arrival.

In a few moments Sir Cooper, accompanied by Lady Perry, was ushered to the platform by Dr. Lauriston Shaw and Dr. Fawcett. At this point, compared with what was to follow, but little demonstration was made, and Dr. Fawcett, as senior physician to the hospital, called upon Dr. Shaw to propose the presentation. Dr. Lauriston Shaw spoke eloquently and at times most delightfully of the days gone by, and of Sir Cooper's brilliant life at Guy's His apt description of the "Guy's community" and of the thousands of others who gained their training in the hospital was fully appreciated by his hearers. They were there to express much more than this poor pen can ever hope to describe, but no one will appreciate their meaning better than the man they were met to honour.

In the absence of Mr. Montagu Hopson, the senior dental surgeon, Dr. Fawcett then formally made the presentation, and Sir Cooper Perry's reply, which, we hope together with the full text of the other speeches to publish next week, was characteristic of the man himself. A delightful speaker, a master of words and their power, and of a keenly receptive nature, he left the men before him with an impression which will live for always in their memory. The more remarkable were the force and strong personality conveyed into Sir Cooper's words, in that to our knowledge he was and had been for some days far from well. Indeed it was up to the last moment doubtful whether he could possibly attend the meeting at all. As he spoke, however, the enthusiasm for the future and the memories of the past, kindled again and again in the hearts of his hearers, and at the close the Court room at Guy's reverberated to such applause as only a truly great man can receive.

As the lane through the packed room opened, cheer upon cheer accompanied Sir Cooper's departure, and so this brief but impressive meeting broke up, a typical demonstration of the great fellowship in Guy's and its universal appreciation of the man who has done so much to further it and the hospital's interests.

**THE PRESENTATION TO SIR E. COOPER PERRY**—(*continued*).

**Address to be Inscribed in Presentation Volume of Signatures.**

GUY'S HOSPITAL.

To SIR EDWIN COOPER PERRY, Kt., M.A., M.D. Cantab., F.R.C.P. Lond.

We whose names are here appended tender you our affectionate regard on the occasion of your retirement from the post of Superintendent to Guy's Hospital.

For thirty years you have devoted a rare and keen intelligence, which has never slumbered, to the fortunes of Guy's.

Coming to us as a physician, with a mind richly endowed with ripe scholarship that has given a peculiar charm to your powers as a clinical teacher, you were very soon called upon to undertake additional duties as an administrator, first as Dean of the Schools and Warden of the College, and later as Superintendent of the Hospital, every department of which, many instituted on your initiative, bears the enduring mark of your singular gifts of practical efficiency.

Upon your courtesy no call has been too heavy. Your help and advice have always been at the disposal of the humblest.

In matters of discipline your decisions have ever been determined by a dominating sense of public duty dedicated to the claims of suffering humanity.

You have maintained and enriched the fame and lustre of the Foundation of Thomas Guy, and given us each and all, professional and laymen alike, an example of high endeavour self-denial and tenacious devotion that we would fain strive to emulate.

We gratefully acknowledge our debt.

January 29, 1920.

